# New insights into the skeletal muscle circadian clock in ruminants

**DOI:** 10.1186/s40104-026-01376-0

**Published:** 2026-03-13

**Authors:** Qiangjun Wang, Yuxin Chen, Yale Chen, Yinghui Ling, Shuai Gao, Zijun Zhang, Dalong Ren

**Affiliations:** 1 College of Animal Science and Technology, Anhui Agricultural University, Hefei, 230036 China; 2 College of Animal Science and Technology, China Agricultural University, Beijing, 100193 China

**Keywords:** Circadian clock, Myogenic differentiation, Ruminants, Satellite cells, Skeletal muscle

## Abstract

Circadian rhythms are endogenous oscillations with a period of approximately 24 h. They enable organisms to anticipate and adapt to daily environmental changes, such as light and temperature. As the largest metabolic and motor organ in the body, skeletal muscle plays a decisive role in determining meat production efficiency in ruminants. Skeletal muscle development is largely governed by the proliferation and myogenic differentiation capacity of skeletal muscle satellite cells (SMSCs). More than 2,300 genes in skeletal muscle exhibit circadian oscillatory expression and are extensively involved in myogenesis, transcriptional regulation, and metabolic processes. The rhythmic expression of these genes is modulated by external factors including the photoperiod, feeding behavior, gut microbiota, and physical activity. Disruption of the endogenous circadian timing system can inhibit SMSC proliferation and myogenic differentiation, thereby impairing normal muscle development. Therefore, this review focuses on key management aspects of ruminant production—such as environmental control, nutritional regulation, and exercise management—and systematically elaborates on how these husbandry strategies may influence SMSC fate by modulating the circadian clock, along with the underlying molecular mechanisms.

## Introduction

Ruminant meat is one of the fastest-growing segments of the global meat market, owing to its high nutritional quality and health benefits. According to the Food and Agriculture Organization, the global populations of cattle, sheep, and goats reached 1.58, 1.32, and 1.13 billion, respectively, in 2023. By 2050, the cattle population is projected to reach 2.6 billion, whereas the combined sheep and goat population is expected to reach 2.7 billion [1]. This expansion underscores the importance of ruminants in the global meat supply. However, ruminant production is more environmentally impactful than non-ruminant production, and its expansion challenges efforts to achieve carbon emission targets [2]. Increasing temperatures, recurrent droughts, and erratic rainfall are increasingly disrupting traditional grazing systems and undermining farm profitability [1]. To address these issues, producers must adopt precision livestock technologies and integrate molecular targeted interventions to increase meat yield [3]. Such integrated approaches are essential for increasing the efficiency and sustainability of ruminant production and for maintaining a stable market supply.

As the largest muscle tissue in ruminants, skeletal muscle development is coordinately regulated by polygenic and nutritional factors that directly influence meat yield and quality [4]. Skeletal muscle originates from myogenic progenitor cells within the embryonic dermomyotome of somites [5]. The proliferation and myogenic differentiation of these cells, which are hierarchically controlled by paired box (PAX) transcription factors, such as *Pax3* and *Pax7*, determine muscle developmental fate [6]. During embryogenesis, PAX3-positive progenitors, stimulated by Wnt and Sonic hedgehog signaling from the notochord, initiate the expression of myogenic regulatory factors (MRFs), including myogenic factor 5 (*Myf5*) and myogenic determining factor (*MyoD*), leading to the formation of primary myofibers [6, 7]. In the late fetal stages, a subset of PAX7-positive cells migrates to the periphery of myofibers, establishing the skeletal muscle satellite cell (SMSC) reservoir [8]. After birth, most SMSCs remain quiescent in the G0 phase [9]. Upon activation by muscle injury or growth signals, SMSCs re-enter the cell cycle via multilevel regulatory mechanisms involving Notch signaling, histone modifications, and miRNAs [10–12]. This activation is marked by *Pax7* downregulation, and *MyoD* and *Myf5* upregulation, which drive myoblast differentiation [13]. Subsequent myogenic differentiation involves sequential activation of *MyoD* and myogenin (*MyoG*), which is coordinated by Wnt/β-catenin signaling, m^6^A RNA methylation, and non-coding RNA networks, which collectively promote myotube formation and myofiber maturation [14–16]. Although the key molecular mechanisms involved in SMSC proliferation and differentiation are known, current knowledge relies heavily on static single-time-point models, limiting insight into spatiotemporal dynamics and the development of precise adaptive strategies for improving meat production efficiency in ruminants.

More than 2,300 protein-coding genes in skeletal muscles exhibit circadian rhythmicity, many of which are involved in myogenesis, muscle repair, and energy metabolism [17]. However, mismanagement of lighting, feeding, exercise, or other husbandry factors can desynchronize the central clock in the suprachiasmatic nucleus (SCN) from peripheral muscle clocks, impairing skeletal muscle growth and development [18]. Key myogenic regulators involved in proliferation (*Pax7*, *MyoD*, and *Myf5*) and differentiation (*MyoD* and *MyoG*) are circadian clock-controlled genes (CCGs), indicating that the circadian clock temporally regulates SMSC fate over 24 h cycles [19]. This review examines how the circadian clock governs SMSC behavior and explores the roles of environmental factors, feeding rhythms, gut microbiota, and physical activity in modulating the muscle circadian system. This study aimed to provide a theoretical foundation and practical insights to support precise health management and enhance the meat productivity of ruminants.

## Circadian clock machinery

### Master circadian clock

**Fig. 1:**
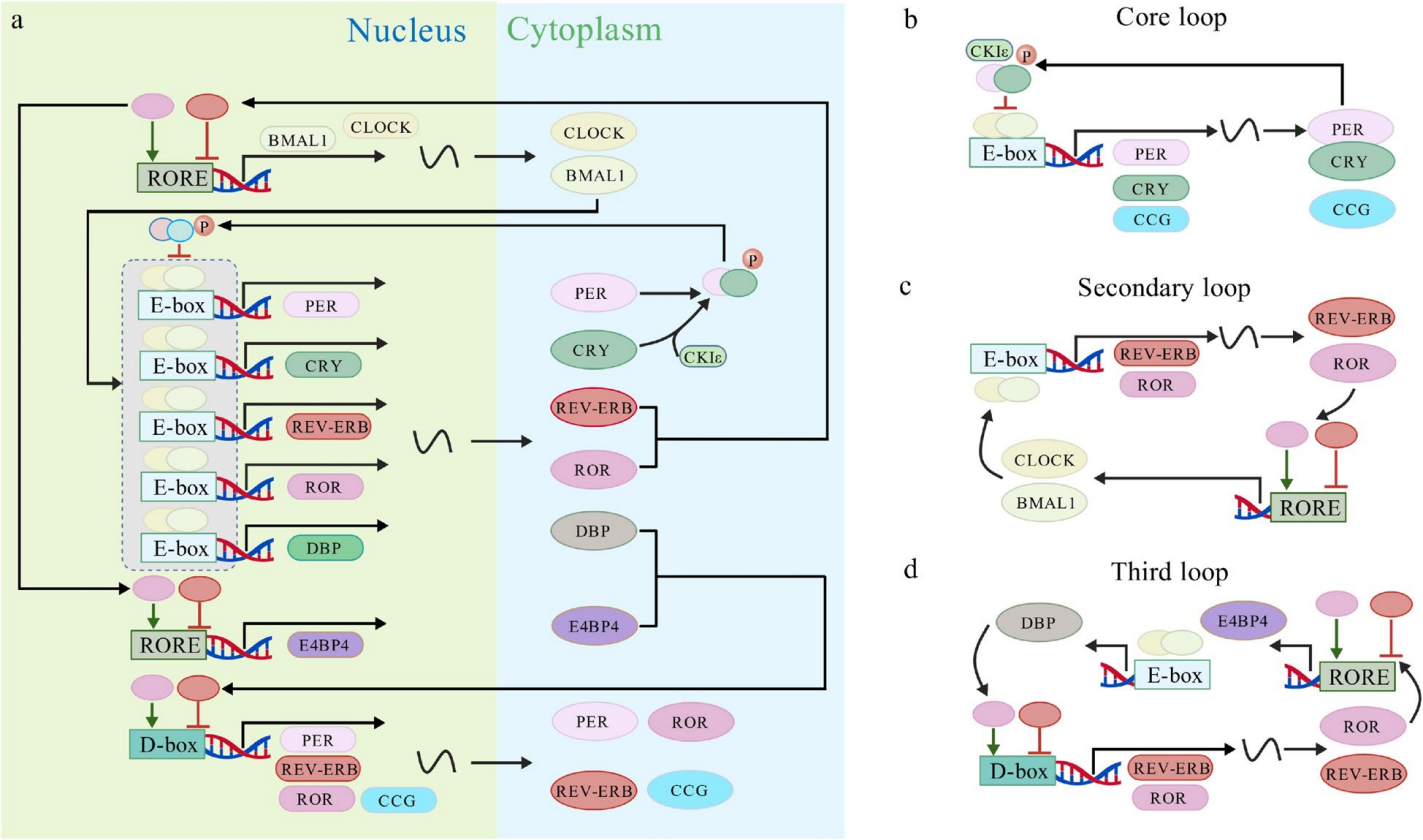
Molecular mechanisms of the circadian clock. **a** Circadian clock feedback loop regulatory network. **b** Core loop. **c** Secondary loop. **d** Third loop. BMAL1, brain and muscle ARNT-like protein 1; CCG, clock-controlled gene; CKIε, casein kinase I epsilon; CLOCK, circadian locomotor output cycles kaput; CRY, cryptochrome; D-box, D-site binding element; DBP, D-site binding protein; E-box, enhance-box; E4BP4, E4-binding protein 4; PER, period; REV-ERB, reverse erb; ROR, retinoic acid receptor-related orphan receptor; RORE, ROR response element

The SCN, the master circadian clock in mammals, synchronizes daily physiological rhythms and facilitates the organism's anticipation and adaptation to environmental cycles, such as light and temperature. This coordination is achieved by regulating gene expression, hormone secretion, and metabolic pathways [20]. At the molecular level, the clock operates via interlocked transcriptional–translational feedback loops (Fig. 1a) [21]. In mammals, the core clock machinery comprises highly conserved genes, including brain and muscle ARNT-like protein 1 (*Bmal1*), circadian locomotor output cycles kaput (*Clock*), period (*Per*), and cryptochrome (*Cry*) [22]. BMAL1 and CLOCK form a heterodimer that binds to E-box enhancer elements and activates the transcription of *Per* and *Cry*. Following translation, PER and CRY translocate to the nucleus and inhibit BMAL1/CLOCK-mediated transcription, forming a core negative feedback loop (Fig. 1b) [23]. In a parallel loop, BMAL1/CLOCK activates transcription of the nuclear receptor reverse erb (REV-ERB) and D-box binding protein (DBP). REV-ERB and retinoic acid-related orphan receptor (ROR) compete for binding to ROR response elements (ROREs), modulating *Clock* and *Bmal1* expression, and establishing a secondary feedback cycle (Fig. 1c) [24]. The third regulatory layer involves competition between E4 promoter-binding protein 4 and DBP for D-box elements, further fine-tuning the circadian output (Fig. 1d) [25]. These interconnected feedback loops, operating through E-box, RORE, and D-box cis-regulatory elements, ensure the robustness and precision of the ~ 24 h circadian cycle [23].

### Peripheral clocks

The core circadian clock components are expressed and functionally active in peripheral tissues, such as the intestine, liver, and skeletal muscle, where their molecular mechanisms remain highly conserved [26–28]. The light‒dark cycle acts as the principal zeitgeber (ZT), synchronizing the central clock in the SCN via photic input through the retina. This central timing signal is then transmitted to peripheral tissues via neural, hormonal, and behavioral pathways, establishing a system-wide circadian network [29, 30]. Importantly, peripheral clocks are entrained by non-photic cues, including feeding-fasting cycles, temperature variations, and physical activity [29]. Desynchronization between the central and peripheral clocks can disrupt metabolic homeostasis, immune function, and tissue development and is linked to pathologies in skeletal muscle, including muscle atrophy, sarcopenia, and muscular dystrophy, underscoring the importance of rhythmicity in muscle maintenance and function [31, 32].

## The molecular clock in SMSC

### Regulation of SMSC proliferation

**Fig. 2:**
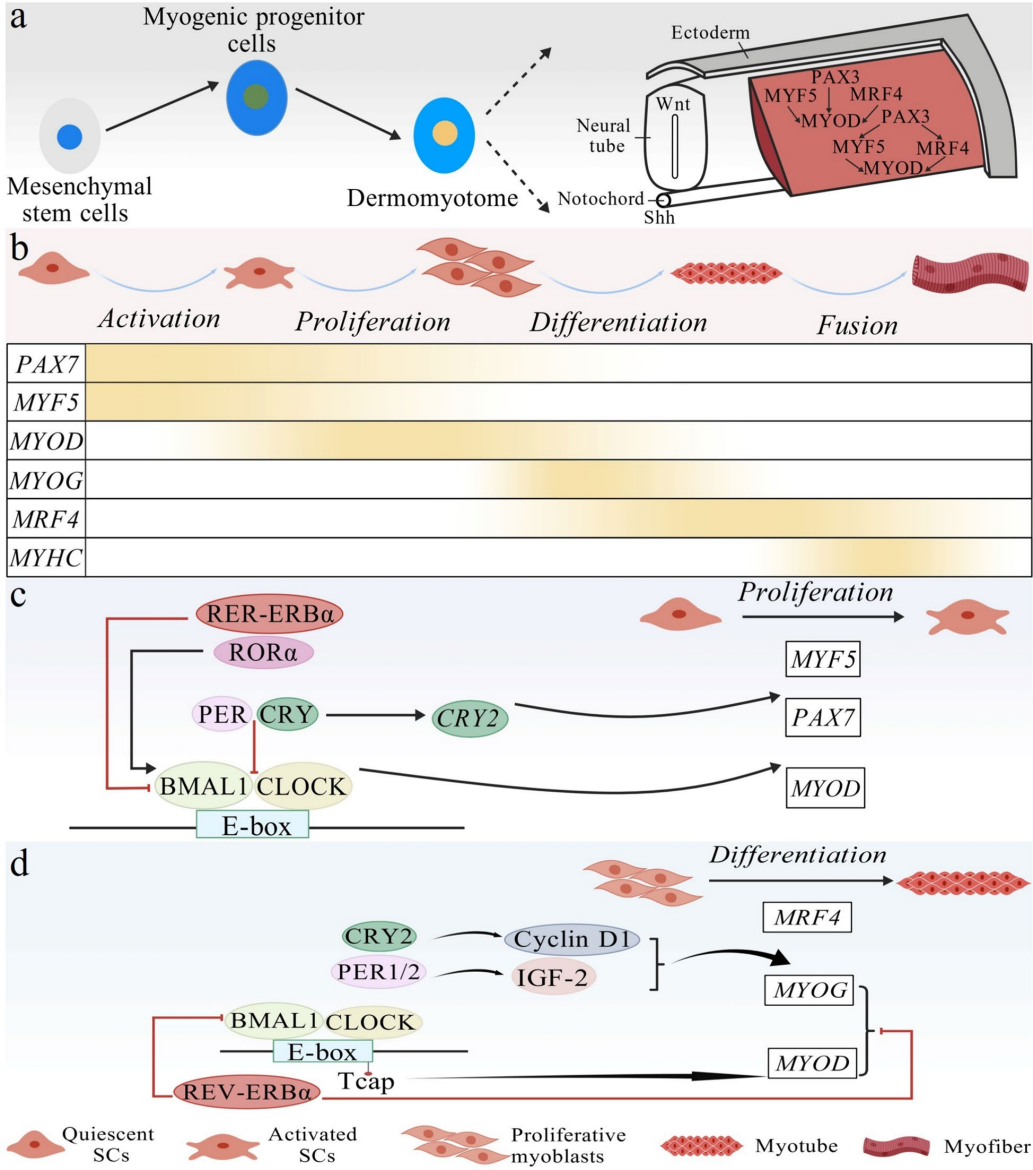
Skeletal muscle development and circadian clock regulation. **a** Mechanisms orchestrating SMSC formation in the embryo. **b** Proliferation and myogenic differentiation of SMSCs during the growth period. **c** Circadian clock regulation of SMSC proliferation. **d** Circadian clock regulation of SMSC differentiation. BMAL1, brain and muscle ARNT-like protein 1; CLOCK, circadian locomotor output cycles kaput; CRY, cryptochrome; E-box, enhance-box; IGF-2, insulin-like growth factor 2; MRF4, myogenic regulatory factor 4; MYF5, myogenic factor 5; MYHC, myosin heavy chain; MYOD, myogenic determining factor; MYOG, myogenin; PAX3, paired box 3; PAX7, paired box 7; PER, period; REV-ERBα, reverse erb alpha; RORα, retinoic acid receptor-related orphan receptor α; SMSC, skeletal muscle satellite cell; Shh, sonic hedgehog; Wnt, wingless-type MMTV integration site family

Mammalian skeletal muscle develops from the paraxial mesoderm, with myogenic progenitors originating in the dorsolateral dermomyotome of somites (Fig. 2a) [5]. After birth, most SMSCs remain quiescent [9]. Activation triggered by local microenvironmental cues involves PAX7-dependent initiation of *Myf5* expression, followed by a decrease in *Pax7* [33]. Subsequently, the activated SMSCs induce *MyoD* expression, re-enter the cell cycle, and commit to the myoblast lineage (Fig. 2b) [13]. SMSC proliferation is precisely controlled by the circadian cycle (Fig. 2c) [19]. The *MyoD* promoter contains E-box elements and directly binds to the CLOCK:BMAL1 heterodimer, leading to rhythmic expression [34, 35]. Similarly, genetic disruption of *Bmal1*^−/−^ or *Clock*^△19^ in mice abolishes *MyoD* mRNA rhythms and impairs their muscle contractility [36]. Furthermore, SMSC proliferation is promoted by *Bmal1* overexpression but is likely suppressed by the PER/CRY complex through the inhibition of CLOCK:BMAL1 transcriptional activity [37]. This model is supported by *Per* knockout studies, in which SMSCs displayed a delayed cell cycle exit [38]. CRY protein degradation relieves transcriptional repression by CLOCK:BMAL1, thereby indirectly enhancing MYOD activity and promoting differentiation [39]. Conversely, *Cry2* knockout increases *Pax7* expression and stimulates proliferation [40]. Among the auxiliary clock pathways, *Ror*^−/−^ leads to impaired motor function and reduced clock gene expression, whereas *Rev-erbα* knockout enhances myogenic gene expression and SMSC proliferation [41, 42]. These effects may be mediated by the ROR-mediated activation and REV-ERB-mediated repression of *BMAL1* transcription, indirectly influencing *MYOD*. However, circadian modulation of SMSC proliferation in ruminants remains largely unexplored. Given the intrinsic and evolutionarily conserved nature of circadian rhythms in regulating cellular functions and that myostatin, a key negative regulator of SMSC proliferation, maintains a phylogenetically conserved circadian regulatory mechanism across species, we hypothesized that the circadian clock governing SMSC proliferation may be evolutionarily conserved across species [43].

### Regulation of myogenic differentiation

The shift from SMSC proliferation to differentiation is characterized by the downregulation of *Pax7* and sustained upregulation of *MyoD* [14]. MYOD activates MYOG and myogenic regulatory factor 4 (MRF4) to initiate myotube formation [14]. Ultimately, MYOD and MYOG cooperate to induce terminal differentiation markers, including *Mrf4* and myosin heavy chain (*MyHC*), thereby driving the assembly of multinucleated myofibers [44, 45]. BMAL1 is a key regulator of this process, enhancing myogenic differentiation by directly upregulating *Myf5* and *MyoD*; modulating Wnt pathway components such as Dishevelled 2, β-catenin, and transcription factor 3; and amplifying Wnt–MRF synergy [46, 47]. Non-ruminant animal models and empirical evidence in goats show that BMAL1-driven transcription of miR-27b suppresses *PAX3*, thereby inhibiting SMSC proliferation [48, 49]. CLOCK:BMAL1-activated MYOD binds to E-box elements in the Tcap promoter, reinforcing differentiation [50]. Although CLOCK:BMAL1 regulates miR-455, which targets *Clock* mRNA, the functional relevance of this feedback mechanism in myogenesis remains unclear [51]. PER1/2 supports myoblast differentiation by binding to the enhancer-promoter regions of insulin-like growth factor 2 (IGF-2), thereby increasing *Igf2* expression and *MyoG* [38]. CRY2 facilitates differentiation and cell fusion by regulating cyclin D1 and Tmem176b, thereby preventing premature cell cycle exit and maintaining *MyoG* levels [52]. In contrast, REV-ERBα acts as a negative regulator; its deletion upregulates *Myf5*, *MyoD*, *MyoG*, and *Wnt3a*, accelerating myotube formation (Fig. 2d) [42]. Several non-coding RNAs influence the fate of SMSCs through specific molecular mechanisms. For example, lncMD1 competitively binds to miR-133a-3p and miR-361-3p, thereby relieving the repression of the target genes *DCTN1*/*DCTN2*, inhibiting proliferation, and promoting differentiation [53]. However, whether the circadian clock modulates SMSCs through non-coding RNA networks remains predominantly unknown. In summary, the core circadian clock negative feedback loop promotes skeletal muscle growth and development by directly or indirectly regulating myogenic differentiation and cell fusion, providing a molecular basis for enhanced meat production.

### Current evidence in ruminants vs. knowledge gaps

Research on the circadian regulation of SMSC proliferation and differentiation has focused on mouse and human models, with limited studies on ruminant models (Table 1). Consistent with non-ruminant model results, *REV-ERBα* overexpression in yaks suppresses SMSC proliferation and differentiation by downregulating the expression of *MYF5*, *MYOD*, and *MYOG* [54]. Studies on sheep and goats have shown that circadian oscillations of clock genes in skeletal muscles correlate with fetal and postnatal growth phases, suggesting a functional link between the muscle clock and developmental physiology [55, 56]. In cattle, *CRY* expression has been associated with meat production traits [57, 58]. However, evidence that the circadian clock directly targets and regulates the proliferation and differentiation of ruminant SMSCs remains scarce. To address these gaps, studies should focus on cross-species comparisons to delineate conserved and species-specific molecular mechanisms of circadian regulation in muscle development.

**Table 1:** Regulatory roles of the circadian clock in skeletal muscle: insights from non-ruminant and ruminant animal models

Clock gene	Non-ruminant models			Ruminant models			References
	Species	Tissue/Cell	Phenotype	Species	Tissue/Cell	Phenotype	
*BMAL1*/*CLOCK*	Mouse; Human	SMSCs	Drives E-box-mediated transcription of *MyoD* to promote muscle growth	Goat; Sheep	Skeletal muscle	Governs skeletal muscle development in fetuses and juveniles; however, the precise mechanism remains unknown	[37, 55, 56, 59]
*PER*/*CRY*	Mouse	SMSCs; Myoblasts	Inhibits CLOCK:BMAL1-mediated transcriptional activation of *MyoD*, thereby suppressing proliferation; *Per* and *Cry2* activate *MyoG* to promote myoblast differentiation	Cattle	Skeletal muscle	*CRY* is associated with carcass traits; however, the molecular mechanism remains unknown	[38, 52, 57, 58]
*REV-ERBα*/*RORα*	Mouse; Human	Myoblasts; Skeletal muscle	*Rev-erbα* inhibits MRF expression to suppress myogenesis; *RORα* promotes BMAL1-mediated transcriptional activation of *MyoD* to facilitate myogenesis	Yak; Goat	SMSCs; Ear tissue	*REV-ERBα* inhibits MRF expression to suppress myogenesis; *RORα* is associated with growth traits; however, the molecular mechanism remains unknown	[41, 42, 54, 60, 61]

## Environmental factors affecting the circadian rhythm of SMSCs

### Photoperiod

**Fig. 3:**
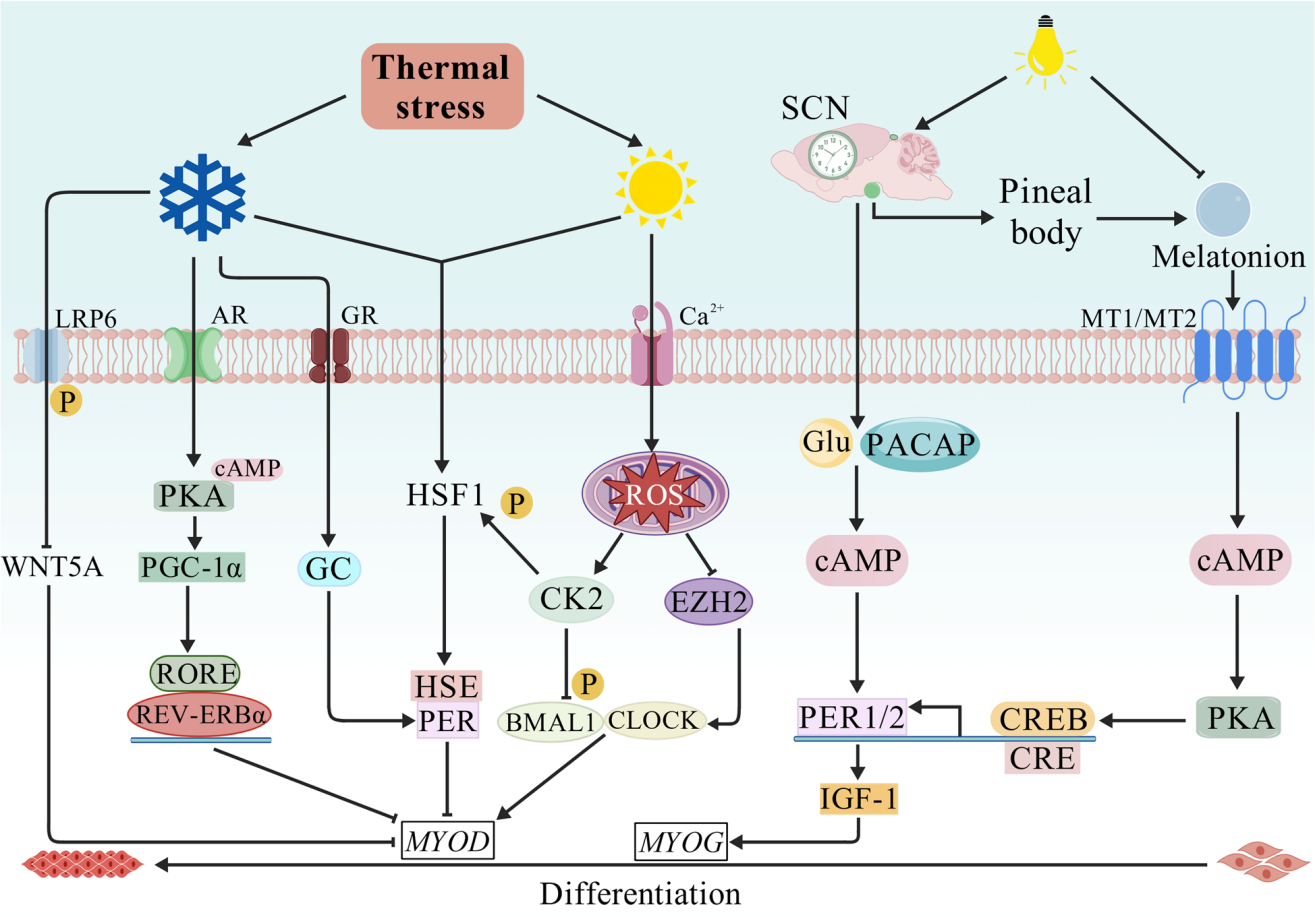
The circadian clock mediates the regulation of skeletal muscle by light and thermal stress. AR, adrenergic receptor; BMAL1, brain and muscle ARNT-like protein 1; cAMP, cyclic adenosine monophosphate; CK2, casein kinase 2; CLOCK, circadian locomotor output cycles kaput; CRE, cAMP response element; CREB, cAMP-response element binding protein; EZH2, enhancer of zeste homolog 2; GC, glucocorticoid; GR, glucocorticoid receptor; HSE, heat shock response element; HSF1, heat shock factor 1; LRP6, low density lipoprotein receptor-related protein 6; MYOD, myogenic determining factor; PACAP, pituitary adenylate cyclase activating polypeptide; PER, period; PKA, protein kinase A; REV-ERBα, reverse erb alpha; RORE, ROR response element; ROS, reactive oxygen species; WNT5A, wingless-type MMTV integration site family member 5A

As the primary ZT, the photoperiod transmits light signals to the SCN via the retina-hypothalamus pathway. The SCN synchronizes peripheral tissue clocks through neural and hormonal signals, establishing systemic circadian coordination (Fig. 3) [62]. *Per1* and *Per2* are central to this process and are key mediators of photic entrainment [63]. Light exposure induces glutamate and pituitary adenylate cyclase-activating polypeptide release in the SCN, increasing intracellular cAMP levels and activating the cAMP-response element binding protein (CREB), which initiates *Per1/2* transcription [64]. This pathway suggests that photoperiod influences ruminant SMSC proliferation and differentiation via circadian regulation. In support of this, extended photoperiods increase average daily gain, carcass weight, and IGF-1 levels in young goats, potentially through PER1/2-mediated activation of IGF-1 signaling and subsequent upregulation of *MYOG* [65]. Conversely, abrupt photoperiod changes disrupt the central–peripheral clock synchrony, reduce muscle *PER1/2* expression, and impair development in sheep [66]. Photoperiod indirectly modulates the clock by regulating melatonin secretion from the pineal gland [67]. Melatonin binds to MT1/MT2 receptors, activates the cAMP-PKA-CREB pathway, and influences *Per1/2* expression [68–70]. In pregnant sows, melatonin supplementation elevates fetal skeletal muscle *PER1/2* expression in the evening, which is concurrent with increased *PAX7* and *MYF5* levels, and enhances myogenic differentiation and birth weight [71]. Although the role of melatonin in ruminant SMSC regulation remains understudied, it may promote proliferation via Wnt pathway-mediated *Pax7* upregulation and myostatin suppression, which is consistent with the observations in lambs [72, 73]. In conclusion, light exposure regulates *PER* expression via the cAMP–CREB pathway, which regulates the proliferation and myogenic differentiation of ruminant SMSCs through CCGs, thus supporting the design of precise lighting regimens to optimize meat production efficiency.

### Thermal environment

Heat stress considerably hinders global meat production, affecting approximately 80% of cattle annually, with > 80% of goats and nearly 60% of sheep reared in hot, arid zones [74, 75]. As a significant ZT, high temperatures can disrupt circadian rhythms, compromising meat production [76]. Mechanistically, heat stress activates heat shock factor 1 (HSF1), which binds to heat shock response elements near the E-box sequences in the *Per2* promoter, potentially desynchronizing the central and peripheral clocks [77, 78]. This dysregulation may inhibit SMSC proliferation and differentiation via CCGs, consistent with the heat-induced downregulation of *PAX7*, *MYOD*, *MYF5*, *MYOG*, and *MYHC* in ovine myoblasts [79]. Heat stress causes direct clock interference and induces reactive oxygen species accumulation, which promotes BMAL1 phosphorylation via casein kinase 2 and disrupts the EZH2–CLOCK/BMAL1 interaction, collectively suppressing MYOD activity and myoblast differentiation [80–82]. Antioxidants, such as resveratrol and vitamin A, mitigate these effects and improve SMSC viability and production performance [83–86]. Cold stress may also impair SMSC function through circadian pathways, potentially via HSF1-PER2-mediated *MyoD* suppression or peroxisome proliferator activated receptor γ coactivator-1α (PGC-1α)-mediated *Rev-erbα* upregulation, which dampens *MyoD* rhythmicity [87–92]. Notably, heat/cold stress disrupts the circadian oscillation of body temperature in mammals, with lamb studies documenting fluctuations reaching 4 °C [93, 94]. In vitro studies employing square-wave temperature cycles to simulate circadian oscillations in body temperature have demonstrated that such thermal fluctuations can reset the circadian clock in myocytes [95]. However, whether extreme climatic conditions affect meat production in ruminants by modulating the circadian clock remains largely unknown. In summary, extreme climatic conditions may disrupt core body temperature and circadian clock oscillations, impair SMSC proliferation and differentiation, and reduce meat production in ruminants. These findings underscore the importance of precisely controlling the thermoneutral environment in ruminant husbandry.

## Nutrition and exercise interfere with the circadian rhythm of SMSCs

### Chrononutrition and feeding time

**Fig. 4:**
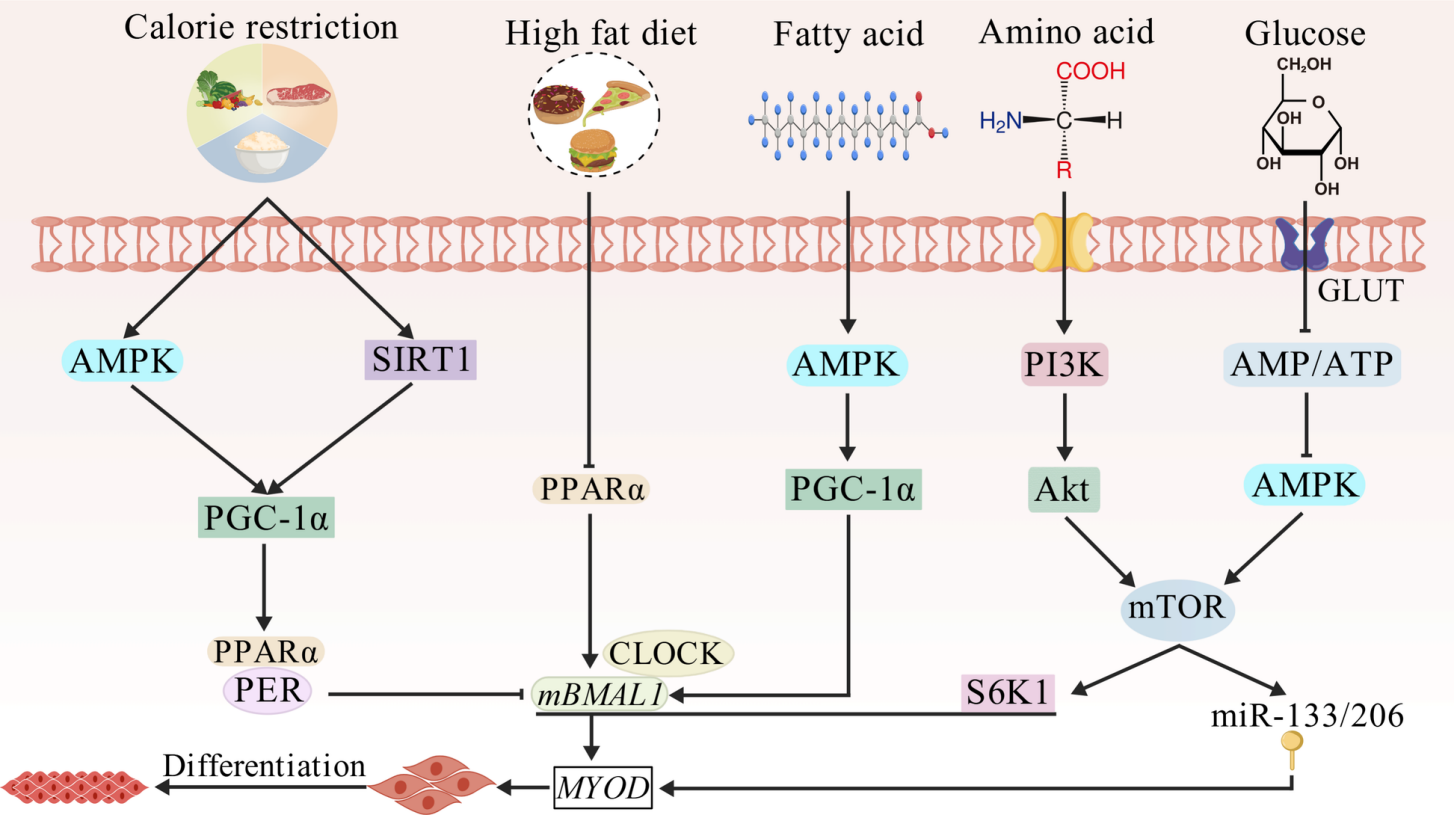
The circadian clock orchestrates nutrient responses in skeletal muscle. Akt, protein kinase B; AMPK, adenosine monophosphate-activated protein kinase; BMAL1, brain and muscle ARNT-like protein 1; CLOCK, circadian locomotor output cycles kaput; mTOR, mammalian target of rapamycin; MYOD, myogenic determining factor; PER, period; PGC-1α, peroxisome proliferator activated receptor γ coactivator-1α; PI3K, phosphatidylinositol 3-kinase; PPARα, peroxisome proliferator activated receptor α; SIRT1, silent information regulator 1; S6K1, ribosomal protein S6 kinase beta-1

Nutrient sensing via the mammalian target of rapamycin (mTOR) pathway regulates myoblast differentiation, whereas miRNAs fine-tune SMSC fate determination (Fig. 4) [96, 97]. Amino acids activate phosphatidylinositol 3-kinase (PI3K)–mTOR–S6K1 signaling, enhance *BMAL1* transcription and MYOD activity, and promote miR-133/206 expression and myoblast differentiation [98]. Physiological glucose uptake suppresses AMPK, derepressing mTOR–BMAL1–MYOD–driven differentiation, whereas excessive glucose activates AMPK, inhibiting mTOR–BMAL1, and impairing differentiation [99, 100]. Fatty acids increase MYOD activity via the AMPK/PGC-1α/BMAL1 axis, whereas high-fat diets suppress CLOCK:BMAL1 DNA-binding and MYOD activity [101, 102]. Caloric restriction activates SIRT1/AMPK/PGC-1α signaling, promoting the PPARα–PER interaction and inhibiting CLOCK:BMAL1, ultimately affecting myogenesis [103, 104]. In *Bmal1*^flox/flox^ mice, breakfast branched-chain amino acid supplementation increased MYF5 activity and muscle hypertrophy, mirroring leucine-induced growth in calves and goats [105–107]. Notably, temporal alignment of feeding with endogenous rhythms enhances myogenesis. Night-restricted feeding in rabbits upregulates *BMAL1*, *CLOCK*, and *MYOG*, and timed protein provision improves piglet growth [95, 108]. Conversely, mistimed feeding disrupts rhythms and promotes adipogenesis [109]. Although data on ruminants remain limited, these findings suggest that macronutrients modulate the circadian clock via intracellular energy sensors to regulate SMSC proliferation and differentiation, whereas disruption of feeding rhythms impairs muscle growth and compromises meat production. Collectively, these results highlight the potential of chrononutrition in increasing production efficiency in ruminant livestock systems.

### The gut microbe–skeletal muscle axis

**Fig. 5:**
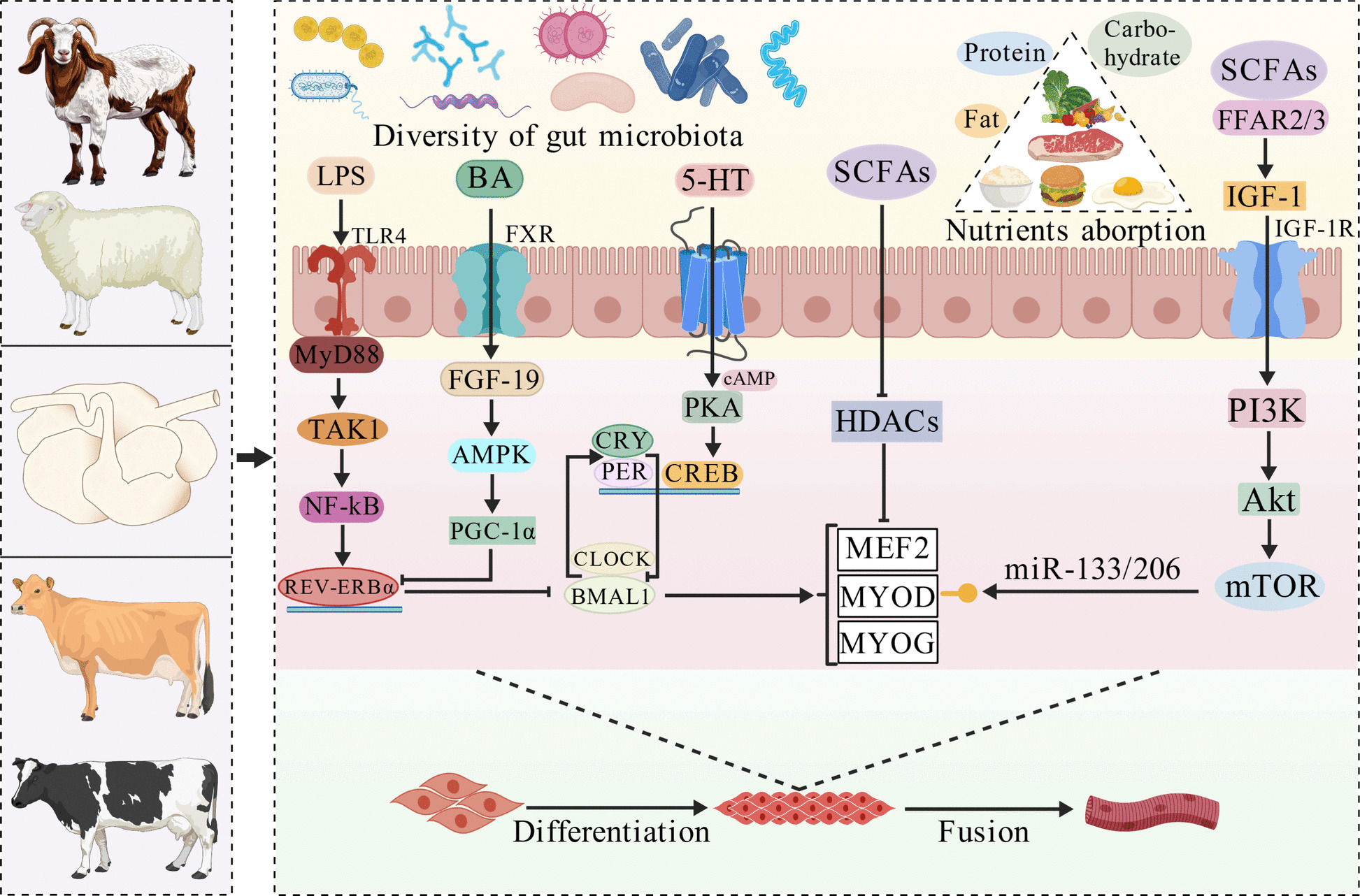
Rumen microbiota regulates skeletal muscle through the circadian clock. Akt, protein kinase B; AMPK, adenosine monophosphate-activated protein kinase; BA, bile acids; BMAL1, brain and muscle ARNT-like protein 1; cAMP, cyclic adenosine monophosphate; CLOCK, circadian locomotor output cycles kaput; CREB, cAMP-response element binding protein; CRY, cryptochrome; FFAR2/3, free fatty acid receptor 2/3; FGF-19, fibroblast growth factor 19; FXR, farnesoid X receptor; HDACs, histone deacetylases; IGF-1, insulin-like growth factor 1; LPS, lipopolysaccharide; MEF2, myocyte enhancer factor 2; mTOR, mammalian target of rapamycin; MyD88, myeloid differentiation primary response 88; MYOD, myogenic determining factor; MYOG, myogenin; NF-kB, nuclear factor kappa-B; PER, period; PGC-1α, peroxisome proliferator activated receptor γ coactivator-1α; PI3K, phosphatidylinositol 3-kinase; PKA, protein kinase A; REV-ERBα, reverse erb alpha; SCFAs, short-chain fatty acids; TAK1, transforming growth factor-β-activated kinase 1; TLR4, Toll-like receptor 4; 5-HT, 5-hydroxytryptamine

The gut microbiota, which exhibits diurnal rhythmicity in ruminants, interacts with the host circadian system (Fig. 5), suggesting its role as a mediator of gut–muscle crosstalk [110, 111]. Microbial metabolites such as short-chain fatty acids (SCFAs) bind to free fatty acid receptor 2/3, promoting IGF-1 release and activating PI3K/Akt/mTOR, potentially enhancing BMAL1–MYOD–miR-133/206 activity and myogenic differentiation [112, 113]. SCFAs inhibit histone deacetylase activity; reduce the acetylation of MYOD, MEF2, and MYOG; and suppress differentiation to maintain SMSC homeostasis [113]. Bile acids activate farnesoid X receptor and FGF19–AMPK/PGC-1α signaling, upregulating myosin, heavy chain 1B, and *PAX7* and increasing myofiber size [114–116]. Furthermore, ruminal microbes that display circadian oscillations are associated with melatonin biosynthesis [117]. Further validation in murine models demonstrated that these microbes modulate intestinal epithelial melatonin synthesis by regulating arylalkylamine N-acetyltransferase activity, suggesting that rumen-derived melatonin may regulate SMSC proliferation and differentiation via *PER1/2* [118]. Conversely, LPS promotes *Rev-erbα* via TLR4–NF-κB, inhibits *MyoD* and differentiation, and triggers muscle atrophy [119–121]. Bacterial serotonin may activate *Per1/2* via GPCR–cAMP–PKA–CREB, thereby inhibiting myogenesis, which is consistent with the pro-regenerative effects of serotonin inhibition [122–124]. Germ-free mice exhibit disrupted clock gene rhythms and muscle atrophy, whereas *Bmal1*^–/–^ mice exhibit microbial rhythm loss, confirming bidirectional host–microbiome interactions [125, 126]. In lambs, *Clostridium butyricum* supplementation improves myofiber size and growth, suggesting that probiotics constitute a promising muscle-enhancing strategy, although circadian involvement requires further study [127]. In summary, rhythmic oscillations in the gut microbiota regulate the SMSC clock via metabolite production, thereby influencing myogenic differentiation and meat production in ruminants through CCGs.

### Chronoexercise

**Fig. 6:**
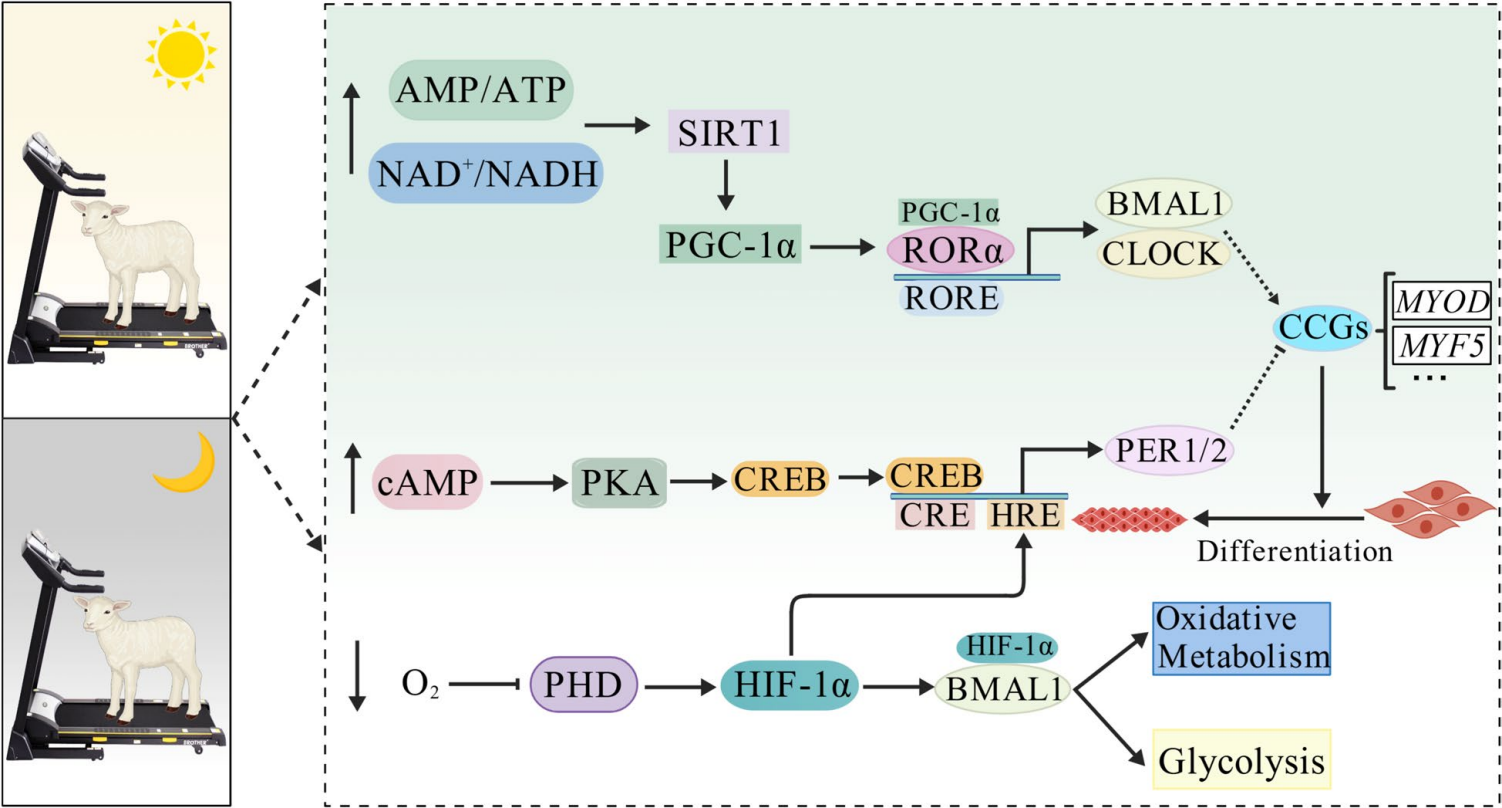
Exercise regulates skeletal muscle through circadian clock-dependent mechanisms. BMAL1, brain and muscle ARNT-like protein 1; cAMP, cyclic adenosine monophosphate; CCGS, clock-controlled genes; CLOCK, circadian locomotor output cycles kaput; CRE, cAMP response element; CREB, cAMP-response element binding protein; HIF-1α, hypoxia-inducible factor 1-alpha; HRE, hypoxia response element; MYF5, myogenic factor 5; MYOD, myogenic determining factor; PER, period; PGC-1α, peroxisome proliferator activated receptor γ coactivator-1α; PHD, prolyl hydroxylase domain; PKA, protein kinase A; RORE, ROR response element; RORα, retinoic acid receptor-related orphan receptor α; SIRT1, silent information regulator 1

Exercise and the circadian clock form a bidirectional regulatory network; core clock genes regulate exercise capacity by modulating metabolism and mitochondrial function, whereas exercise feedback adjusts the clock amplitude/phase and influences the muscle phenotype via CCGs (Fig. 6) [128–130]. Exercise-induced ATP/NADH depletion activates AMPK/SIRT1 and modulates PER2/CRY1 stability and clock periodicity [131]. Exercise upregulates *PGC-1α*, enhances mitochondrial biogenesis, and activates RORα/γ to reinforce BMAL1/CLOCK transcription and myofiber-type transition [132]. HIF-1α modulation integrates hypoxic and circadian signals, optimizing metabolic balance [133]. Through these pathways, exercise shifts the core clock gene expression, regulates protein turnover, activates SMSCs, and induces hypertrophy, collectively supporting muscle development. Grazing improves lamb finishing performance compared with that in indoor systems, and gestational exercise in cattle increases the number of offspring myofibers [134, 135]. The efficacy of such strategies is chronodependent, with afternoon exercises generally optimizing outcomes [136]. Thus, chronoexercise enhances the circadian expression of the SMSC clock by modulating energy metabolism and reinforcing the circadian amplitude/phase, which subsequently optimizes muscle fiber type composition through CCGs and promotes skeletal muscle growth. This mechanism provides a viable exercise management strategy to improve meat production in ruminants.

## Conclusion

The circadian clock precisely regulates SMSC proliferation and differentiation in ruminants and is influenced by photoperiod, temperature, feeding patterns, gut microbiota, and exercise. Although multifactorial interactions remain understudied, these factors form interconnected networks. The gut microbiota mediates host rhythms and muscle development and responds to environmental and dietary cues, necessitating a deeper mechanistic insight. Elucidating clock–SMSC interactions will refine management and nutritional strategies, support muscle growth, and facilitate precision health farming. Future studies should dissect external factor–clock–muscle interactions to sustainably enhance meat production.

## Data Availability

Not applicable.

## Abbreviations

Bmal1: Brain and muscle ARNT-like protein 1
CCGs: Clock-controlled genes
Clock: Circadian locomotor output cycles kaput
CREB: CAMP-response element binding protein
Cry: Cryptochrome
E-box: Enhance-box
HSF1: Heat shock factor 1
IGF: Insulin-like growth factor
MRF4: Myogenic regulatory factor 4
MRFs: Myogenic regulatory factors
mTOR: Mammalian target rapamycin
Myf5: Myogenic factor 5
MyHC: Myosin heavy chain
MyoD: Myogenic determining factor
MyoG: Myogenin
PAX: Paired box
Per: Period
PGC-1α: Peroxisome proliferator activated receptor γ coactivator-1α
PI3K: Phosphatidylinositol 3-kinase
REV-ERB: Reverse erb
RORE: ROR response element
RORα: Retinoic acid receptor-related orphan receptor α
SCFAs: Short-chain fatty acids
SCN: Suprachiasmatic nucleus
SIRT1: Silent information regulator 1
SMSCs: Skeletal muscle satellite cells
Wnt: Wingless-type MMTV integration site family
ZT: Zeitgeber

## References

[1] Turk J. Meeting projected food demands by 2050: understanding and enhancing the role of grazing ruminants. J Anim Sci. 2016;94(6):53–62. 10.2527/jas.2016-0547.

[2] Du Y, Ge Y, Chang J. Global strategies to minimize environmental impacts of ruminant production. Annu Rev Anim Biosci. 2022;10:227–40. 10.1146/annurev-animal-020420-043152. 34780247 10.1146/annurev-animal-020420-043152

[3] Du Y, Ge Y, Ren Y, Fan X, Pan K, Lin L, et al. A global strategy to mitigate the environmental impact of China’s ruminant consumption boom. Nat Commun. 2018;9:4133. 10.1038/s41467-018-06381-0. 10.1038/s41467-018-06381-0 PMC6175953 30297840

[4] Ling YH, Zheng Q, Jing J, Sui MH, Zhu L, Li YS, et al. Switches in transcriptome functions during seven skeletal muscle development stages from fetus to kid in *Capra hircus*. J Integr Agric. 2021;20(1):212–26. 10.1016/S2095-3119(20)63268-3.

[5] Zhang W, Liu Y, Zhang H. Extracellular matrix: an important regulator of cell functions and skeletal muscle development. Cell Biosci. 2021;11(1):65. 10.1186/s13578-021-00579-4. 33789727 10.1186/s13578-021-00579-4 PMC8011170

[6] Bou Akar R, Lama C, Aubin D, Maruotti J, Onteniente B, de Esteves Lima J, et al. Generation of highly pure pluripotent stem cell-derived myogenic progenitor cells and myotubes. Stem Cell Rep. 2024;19(1):84–99. 10.1016/j.stemcr.2023.11.002. 10.1016/j.stemcr.2023.11.002 PMC10828960 38101399

[7] Magli A, Perlingeiro RRC. Myogenic progenitor specification from pluripotent stem cells. Semin Cell Dev Biol. 2017;72:87–98. 10.1016/j.semcdb.2017.10.031. 29107681 10.1016/j.semcdb.2017.10.031 PMC5723218

[8] Fung CW, Zhou S, Zhu H, Wei X, Wu Z, Wu AR. Cell fate determining molecular switches and signaling pathways in Pax7-expressing somitic mesoderm. Cell Discov. 2022;8(1):61. 10.1038/s41421-022-00407-0. 35764624 10.1038/s41421-022-00407-0 PMC9240041

[9] Campanario S, Ramírez-Pardo I, Hong X, Isern J, Muñoz-Cánoves P. Assessing autophagy in muscle stem cells. Front Cell Dev Biol. 2020;8:620409. 10.3389/fcell.2020.620409. 33553156 10.3389/fcell.2020.620409 PMC7858272

[10] Gonzalez ML, Busse NI, Waits CM, Johnson SE. Satellite cells and their regulation in livestock. J Anim Sci. 2020;98(5):skaa081. 10.1093/jas/skaa081. 32175577 10.1093/jas/skaa081 PMC7193651

[11] Boonsanay V, Zhang T, Georgieva A, Kostin S, Qi H, Yuan X, et al. Regulation of skeletal muscle stem cell quiescence by Suv4-20h1-dependent facultative heterochromatin formation. Cell Stem Cell. 2016;18(2):229–42. 10.1016/j.stem.2015.11.002. 26669898 10.1016/j.stem.2015.11.002

[12] Stepicheva NA, Song JL. Function and regulation of microRNA-31 in development and disease. Mol Reprod Dev. 2016;83(8):654–74. 10.1002/mrd.22678. 27405090 10.1002/mrd.22678 PMC6040227

[13] VanGenderen CA, Granet JA, Filippelli RL, Liu Y, Chang NC. Modulating myogenesis: an optimized in vitro assay to pharmacologically influence primary myoblast differentiation. Curr Protoc. 2022;2(9):e565. 10.1002/cpz1.565. 36165685 10.1002/cpz1.565

[14] Pajalunga D, Crescenzi M. Restoring the cell cycle and proliferation competence in terminally differentiated skeletal muscle myotubes. Cells. 2021;10(10):2753. 10.3390/cells10102753. 34685732 10.3390/cells10102753 PMC8534385

[15] Cui S, Li L, Yu RT, Downes M, Evans RM, Hulin JA, et al. β-Catenin is essential for differentiation of primary myoblasts via cooperation with MyoD and α-catenin. Development. 2019;146(6):dev167080. 10.1242/dev.167080. 30683662 10.1242/dev.167080 PMC6451316

[16] Chen SL, Wu CC, Li N, Weng TH. Post-transcriptional regulation of myogenic transcription factors during muscle development and pathogenesis. J Muscle Res Cell Motil. 2024;45(1):21–39. 10.1007/s10974-023-09663-3. 38206489 10.1007/s10974-023-09663-3

[17] Zhang R, Lahens NF, Ballance HI, Hughes ME, Hogenesch JB. A circadian gene expression atlas in mammals: implications for biology and medicine. Proc Natl Acad Sci U S A. 2014;111(45):16219–24. 10.1073/pnas.1408886111. 25349387 10.1073/pnas.1408886111 PMC4234565

[18] Kim HK, Shibata S. Circadian rhythm and muscle function. Adv Exp Med Biol. 2025;1478:101–12. 10.1007/978-3-031-88361-3_6. 40879938 10.1007/978-3-031-88361-3_6

[19] Kahn RE, Dayanidhi S, Lacham-Kaplan O, Hawley JA. Molecular clocks, satellite cells, and skeletal muscle regeneration. Am J Physiol Cell Physiol. 2023;324(6):C1332–40. 10.1152/ajpcell.00073.2023. 37184229 10.1152/ajpcell.00073.2023 PMC11932531

[20] Laothamatas I, Rasmussen ES, Green CB, Takahashi JS. Metabolic and chemical architecture of the mammalian circadian clock. Cell Chem Biol. 2023;30(9):1033–52. 10.1016/j.chembiol.2023.08.014. 37708890 10.1016/j.chembiol.2023.08.014 PMC10631358

[21] Yi JS, Díaz NM, D’Souza S, Buhr ED. The molecular clockwork of mammalian cells. Semin Cell Dev Biol. 2022;126:87–96. 10.1016/j.semcdb.2021.03.012. 33810978 10.1016/j.semcdb.2021.03.012 PMC8481349

[22] Patke A, Young MW, Axelrod S. Molecular mechanisms and physiological importance of circadian rhythms. Nat Rev Mol Cell Biol. 2019;21(2):67–84. 10.1038/s41580-019-0179-2. 31768006 10.1038/s41580-019-0179-2

[23] Partch CL, Green CB, Takahashi JS. Molecular architecture of the mammalian circadian clock. Trends Cell Biol. 2014;24(2):90–9. 10.1016/j.tcb.2013.07.002. 23916625 10.1016/j.tcb.2013.07.002 PMC3946763

[24] Cho H, Zhao X, Hatori M, Yu RT, Barish GD, Lam MT, et al. Regulation of circadian behaviour and metabolism by REV-ERB-α and REV-ERB-β. Nature. 2012;485(7396):123–7. 10.1038/nature11048. 22460952 10.1038/nature11048 PMC3367514

[25] Kim YH, Lazar MA. Transcriptional control of circadian rhythms and metabolism: a matter of time and space. Endocr Rev. 2020;41(5):707–32. 10.1210/endrev/bnaa014. 32392281 10.1210/endrev/bnaa014 PMC7334005

[26] Hibberd TJ, Ramsay S, Spencer-Merris P, Dinning PG, Zagorodnyuk VP, Spencer NJ. Circadian rhythms in colonic function. Front Physiol. 2023;14:1239278. 10.3389/fphys.2023.1239278. 37711458 10.3389/fphys.2023.1239278 PMC10498548

[27] Koronowski KB, Kinouchi K, Welz PS, Smith JG, Zinna VM, Shi J, et al. Defining the independence of the liver circadian clock. Cell. 2019;177(6):1448-62.e14. 10.1016/j.cell.2019.04.025. 31150621 10.1016/j.cell.2019.04.025 PMC6813833

[28] Smith JG, Koronowski KB, Mortimer T, Sato T, Greco CM, Petrus P, et al. Liver and muscle circadian clocks cooperate to support glucose tolerance in mice. Cell Rep. 2023;42(6):112588. 10.1016/j.celrep.2023.112588. 37267101 10.1016/j.celrep.2023.112588 PMC10592114

[29] Zhang S, Dai M, Wang X, Jiang SH, Hu LP, Zhang XL, et al. Signalling entrains the peripheral circadian clock. Cell Signal. 2020;69:109433. 10.1016/j.cellsig.2019.109433. 31982551 10.1016/j.cellsig.2019.109433

[30] Dibner C, Schibler U, Albrecht U. The mammalian circadian timing system: organization and coordination of central and peripheral clocks. Annu Rev Physiol. 2010;72:517–49. 10.1146/annurev-physiol-021909-135821. 20148687 10.1146/annurev-physiol-021909-135821

[31] Bautista J, Ojeda-Mosquera S, Ordóñez-Lozada D, López-Cortés A. Peripheral clocks and systemic zeitgeber interactions: from molecular mechanisms to circadian precision medicine. Front Endocrinol (Lausanne). 2025;16:1606242. 10.3389/fendo.2025.1606242. 40510487 10.3389/fendo.2025.1606242 PMC12158691

[32] Kiperman T, Ma K. Circadian clock in muscle disease etiology and therapeutic potential for duchenne muscular dystrophy. Int J Mol Sci. 2024;25(9):4767. 10.3390/ijms25094767. 38731986 10.3390/ijms25094767 PMC11083552

[33] Zammit PS. Function of the myogenic regulatory factors Myf5, MyoD, Myogenin and MRF4 in skeletal muscle, satellite cells and regenerative myogenesis. Semin Cell Dev Biol. 2017;72:19–32. 10.1016/j.semcdb.2017.11.011. 29127046 10.1016/j.semcdb.2017.11.011

[34] Andrews JL, Zhang X, McCarthy JJ, McDearmon EL, Hornberger TA, Russell B, et al. CLOCK and BMAL1 regulate MyoD and are necessary for maintenance of skeletal muscle phenotype and function. Proc Natl Acad Sci U S A. 2010;107(44):19090–5. 10.1073/pnas.1014523107. 20956306 10.1073/pnas.1014523107 PMC2973897

[35] Zhang X, Patel SP, McCarthy JJ, Rabchevsky AG, Goldhamer DJ, Esser KA. A non-canonical E-box within the MyoD core enhancer is necessary for circadian expression in skeletal muscle. Nucleic Acids Res. 2012;40(8):3419–30. 10.1093/nar/gkr1297. 22210883 10.1093/nar/gkr1297 PMC3333858

[36] Harfmann BD, Schroder EA, Esser KA. Circadian rhythms, the molecular clock, and skeletal muscle. J Biol Rhythms. 2015;30(2):84–94. 10.1177/0748730414561638. 25512305 10.1177/0748730414561638 PMC4470613

[37] Chatterjee S, Yin H, Nam D, Li Y, Ma K. Brain and muscle Arnt-like 1 promotes skeletal muscle regeneration through satellite cell expansion. Exp Cell Res. 2015;331(1):200–10. 10.1016/j.yexcr.2014.08.041. 25218946 10.1016/j.yexcr.2014.08.041

[38] Katoku-Kikyo N, Paatela E, Houtz DL, Lee B, Munson D, Wang X, et al. Per1/Per2-Igf2 axis-mediated circadian regulation of myogenic differentiation. J Cell Biol. 2021;220(7):e202101057. 10.1083/jcb.202101057. 34009269 10.1083/jcb.202101057 PMC8138781

[39] Blondelle J, Biju A, Lange S. The role of cullin-RING ligases in striated muscle development, function, and disease. Int J Mol Sci. 2020;21(21):7936. 10.3390/ijms21217936. 33114658 10.3390/ijms21217936 PMC7672578

[40] Hao Y, Xue T, Liu SB, Geng S, Shi X, Qian P, et al. Loss of CRY2 promotes regenerative myogenesis by enhancing PAX7 expression and satellite cell proliferation. MedComm. 2023;4(1):e202. 10.1002/mco2.202. 36636367 10.1002/mco2.202 PMC9830134

[41] Mansingh S, Maier G, Delezie J, Westermark PO, Ritz D, Duchemin W, et al. More than the clock: distinct regulation of muscle function and metabolism by PER2 and RORα. J Physiol. 2024;602(23):6373–402. 10.1113/jp285585. 38850551 10.1113/JP285585 PMC11607892

[42] Chatterjee S, Yin H, Li W, Lee J, Yechoor VK, Ma K. The nuclear receptor and clock repressor Rev-erbα suppresses myogenesis. Sci Rep. 2019;9:4585. 10.1038/s41598-019-41059-7. 30872796 10.1038/s41598-019-41059-7 PMC6418265

[43] Liu X, Song C, Xiong Y, Gu J, Wu L, Liu T, et al. Myostatin exhibits an evolutionarily conserved circadian pattern in skeletal muscles. J Cachexia Sarcopenia Muscle. 2025;16(6):e70130. 10.1002/jcsm.70130. 41287261 10.1002/jcsm.70130 PMC12644245

[44] Hernández-Hernández JM, García-González EG, Brun CE, Rudnicki MA. The myogenic regulatory factors, determinants of muscle development, cell identity and regeneration. Semin Cell Dev Biol. 2017;72:10–8. 10.1016/j.semcdb.2017.11.010. 29127045 10.1016/j.semcdb.2017.11.010 PMC5723221

[45] Pang KT, Loo LSW, Chia S, Ong FYT, Yu H, Walsh I. Insight into muscle stem cell regeneration and mechanobiology. Stem Cell Res Ther. 2023;14(1):129. 10.1186/s13287-023-03363-y. 37173707 10.1186/s13287-023-03363-y PMC10176686

[46] Hodge BA, Zhang X, Gutierrez-Monreal MA, Cao Y, Hammers DW, Yao Z, et al. MYOD1 functions as a clock amplifier as well as a critical co-factor for downstream circadian gene expression in muscle. Elife. 2019;8:e43017. 10.7554/eLife.43017. 30789342 10.7554/eLife.43017 PMC6398978

[47] Zhu P, Hamlish NX, Thakkar AV, Steffeck AWT, Rendleman EJ, Khan NH, et al. BMAL1 drives muscle repair through control of hypoxic NAD^+^ regeneration in satellite cells. Genes Dev. 2022;36(3–4):149–66. 10.1101/gad.349066.121. 35115380 10.1101/gad.349066.121 PMC8887128

[48] Wang H, Fan Z, Zhao M, Li J, Lu M, Liu W, et al. Oscillating primary transcripts harbor miRNAs with circadian functions. Sci Rep. 2016;6:21598. 10.1038/srep21598. 26898952 10.1038/srep21598 PMC4761921

[49] Ling YH, Sui MH, Zheng Q, Wang KY, Wu H, Li WY, et al. miR-27b regulates myogenic proliferation and differentiation by targeting Pax3 in goat. Sci Rep. 2018;8:3909. 10.1038/s41598-018-22262-4. 10.1038/s41598-018-22262-4 PMC5834623 29500394

[50] Cao X, Yan Y, Luo X, Yang X, Cui H, Yang Y, et al. Analyses of the circadian clock genes expression in whole embryos and maternal major tissues of mice. J Mol Histol. 2022;53(2):473–82. 10.1007/s10735-022-10065-x. 35149920 10.1007/s10735-022-10065-x

[51] Cheng Q, Fan X, Liu Y, Xu L, Dong P, Song L, et al. miR-455-5p regulates circadian rhythms by accelerating the degradation of Clock mRNA. IUBMB Life. 2022;74(3):245–58. 10.1002/iub.2587. 34904778 10.1002/iub.2587

[52] Lowe M, Lage J, Paatela E, Munson D, Hostager R, Yuan C, et al. Cry2 is critical for circadian regulation of myogenic differentiation by Bclaf1-mediated mRNA stabilization of cyclin D1 and Tmem176b. Cell Rep. 2018;22(8):2118–32. 10.1016/j.celrep.2018.01.077. 29466738 10.1016/j.celrep.2018.01.077 PMC5849083

[53] Jing J, Yang WX, Pan QQ, Zhang SH, Cao HG, Zhang ZJ, et al. Regulatory role of lncMD1 in goat skeletal muscle satellite cell differentiation via miR-133a-3p and miR-361-3p targeting. Int J Biol Macromol. 2024;280:135807. 10.1016/j.ijbiomac.2024.135807. 39306179 10.1016/j.ijbiomac.2024.135807

[54] Zhe Y, Wu Z, Yasenjian S, Zhong J, Jiang H, Zhang M, et al. Effect of NR1D1 on the proliferation and differentiation of yak skeletal muscle satellite cells. Front Vet Sci. 2024;11:1428117. 10.3389/fvets.2024.1428117. 39559540 10.3389/fvets.2024.1428117 PMC11571325

[55] Zhou X, Yan Q, Yang H, Ren A, He Z, Tan Z. Maternal intake restriction programs the energy metabolism, clock circadian regulator and mTOR signals in the skeletal muscles of goat offspring probably via the protein kinase A-cAMP-responsive element-binding proteins pathway. Anim Nutr. 2021;7(4):1303–14. 10.1016/j.aninu.2021.09.006. 34786503 10.1016/j.aninu.2021.09.006 PMC8567324

[56] Gatford KL, Kennaway DJ, Liu H, Kleemann DO, Kuchel TR, Varcoe TJ. Simulated shift work disrupts maternal circadian rhythms and metabolism, and increases gestation length in sheep. J Physiol. 2019;597(7):1889–904. 10.1113/jp277186. 30671970 10.1113/JP277186 PMC6441904

[57] Zhang K, Mi F, Li X, Wang Z, Jiang F, Song E, et al. Detection of genetic variation in bovine CRY1 gene and its associations with carcass traits. Anim Biotechnol. 2022;34(8):3387–94. 10.1080/10495398.2022.2149547. 36448652 10.1080/10495398.2022.2149547 PMC13353454

[58] Li X, Jiang E, Zhang K, Zhang S, Jiang F, Song E, et al. Genetic variations within the bovine CRY2 gene are significantly associated with carcass traits. Animals. 2022;12(13):1616. 10.3390/ani12131616. 35804515 10.3390/ani12131616 PMC9264869

[59] Figueroa-Toledo AM, Gutiérrez-Pino J, Carriel-Nesvara A, Marchese-Bittencourt M, Zbinden-Foncea H, Castro-Sepúlveda M. BMAL1 and CLOCK proteins exhibit differential association with mitochondrial dynamics, protein synthesis pathways and muscle strength in human muscle. J Physiol. 2024;602(23):6403–15. 10.1113/jp285955. 38922907 10.1113/JP285955

[60] Wei L, Yue Q, Yang D, Miao S, Lu M, Zhang Z, et al. The retinoic acid-related orphan receptors (RORs) signaling axis in skeletal homeostasis: mechanistic insights and chronotherapeutic prospects. Ann Med. 2026;58(1):2615534. 10.1080/07853890.2026.2615534. 41530944 10.1080/07853890.2026.2615534 PMC12805853

[61] Zhou Q, Hu H, Yang Y, Kang Y, Lan X, Wu X, et al. Insertion/deletion (Indel) variant of the goat RORA gene is associated with growth traits. Anim Biotechnol. 2022;34(7):2175–82. 10.1080/10495398.2022.2078980. 35622416 10.1080/10495398.2022.2078980

[62] Astiz M, Heyde I, Oster H. Mechanisms of communication in the mammalian circadian timing system. Int J Mol Sci. 2019;20(2):343. 10.3390/ijms20020343. 30650649 10.3390/ijms20020343 PMC6359556

[63] Sato RY, Yamanaka Y. Nonphotic entrainment of central and peripheral circadian clocks in mice by scheduled voluntary exercise under constant darkness. Am J Physiol Regul Integr Comp Physiol. 2023;324(4):R526–35. 10.1152/ajpregu.00320.2022. 36802951 10.1152/ajpregu.00320.2022

[64] Ashton A, Foster RG, Jagannath A. Photic entrainment of the circadian system. Int J Mol Sci. 2022;23(2):729. 10.3390/ijms23020729. 35054913 10.3390/ijms23020729 PMC8775994

[65] Vélez LI, Flores MJ, Hernández H, Vargas-Cruz AA, Avilés R, Rosales-Nieto CA. Artificial long-day photoperiod enhances growth performance and metabolic indicators in young male goats. J Anim Sci. 2025;103:skaf270. 10.1093/jas/skaf270. 40795883 10.1093/jas/skaf270 PMC12448396

[66] Varcoe TJ, Gatford KL, Voultsios A, Salkeld MD, Boden MJ, Rattanatray L, et al. Rapidly alternating photoperiods disrupt central and peripheral rhythmicity and decrease plasma glucose, but do not affect glucose tolerance or insulin secretion in sheep. Exp Physiol. 2014;99(9):1214–28. 10.1113/expphysiol.2014.080630. 24951500 10.1113/expphysiol.2014.080630

[67] Ren L, Okimura K, Ishikawa A, Kon N, Shimba S, Yoshimura T. The role of circadian clock gene Arntl in the winter depression-like behavior in melatonin-proficient female CBA/N mice. Biochem Biophys Res Commun. 2024;734:150790. 10.1016/j.bbrc.2024.150790. 39369541 10.1016/j.bbrc.2024.150790

[68] Rodríguez-Santana C, Florido J, Martínez-Ruiz L, López-Rodríguez A, Acuña-Castroviejo D, Escames G. Role of melatonin in cancer: effect on clock genes. Int J Mol Sci. 2023;24(3):1919. 10.3390/ijms24031919. 36768253 10.3390/ijms24031919 PMC9916653

[69] Li SJ, Cheng WL, Kao YH, Chung CC, Trang NN, Chen YJ. Melatonin inhibits NF-κB/CREB/Runx2 signaling and alleviates aortic valve calcification. Front Cardiovasc Med. 2022;9:885293. 10.3389/fcvm.2022.885293. 35795373 10.3389/fcvm.2022.885293 PMC9251177

[70] Liu Y, Ni C, Li Z, Yang N, Zhou Y, Rong X, et al. Prophylactic melatonin attenuates isoflurane-induced cognitive impairment in aged rats through hippocampal melatonin receptor 2-cAMP response element binding signalling. Basic Clin Pharmacol Toxicol. 2017;120(3):219–26. 10.1111/bcpt.12652. 27515785 10.1111/bcpt.12652

[71] Dobbins TW, Swanson RM, Dennis AA, Rivera JD, Dinh TTN, Lemley CO, et al. Melatonin supplementation to sows in mid to late gestation affects offspring circadian, myogenic, and growth factor transcript abundance in pre and postnatal skeletal muscle. J Anim Sci. 2024;102:skae377. 10.1093/jas/skae377. 39679952 10.1093/jas/skae377 PMC11683838

[72] Su CM, Tsai CH, Chen HT, Wu YS, Chang JW, Yang SF, et al. Melatonin improves muscle injury and differentiation by increasing Pax7 expression. Int J Biol Sci. 2023;19(4):1049–62. 10.7150/ijbs.79169. 36923937 10.7150/ijbs.79169 PMC10008686

[73] Ma W, Wu H, Li G, Yan L, Wang L, Zhao M, et al. Melatonin promotes the growth and development of lambs by increasing growth hormone and testosterone, targeting on apoptosis signaling pathway and intestinal microflora. Front Endocrinol (Lausanne). 2022;13:966120. 10.3389/fendo.2022.966120. 36060949 10.3389/fendo.2022.966120 PMC9439620

[74] North MA, Franke JA, Ouweneel B, Trisos CH. Global risk of heat stress to cattle from climate change. Environ Res Lett. 2023;18(9):094027. 10.1088/1748-9326/aceb79.

[75] Joy A, Dunshea FR, Leury BJ, Clarke IJ, DiGiacomo K, Chauhan SS. Resilience of small ruminants to climate change and increased environmental temperature: a review. Animals. 2020;10(5):867. 10.3390/ani10050867. 32429527 10.3390/ani10050867 PMC7278399

[76] Refinetti R. Circadian rhythmicity of body temperature and metabolism. Temperature. 2020;7(4):321–62. 10.1080/23328940.2020.1743605. 10.1080/23328940.2020.1743605 PMC7678948 33251281

[77] Kawamura G, Hattori M, Takamatsu K, Tsukada T, Ninomiya Y, Benjamin I, et al. Cooperative interaction among BMAL1, HSF1, and p53 protects mammalian cells from UV stress. Commun Biol. 2018;1:204. 10.1038/s42003-018-0209-1. 30480104 10.1038/s42003-018-0209-1 PMC6250677

[78] Tamaru T, Hattori M, Honda K, Benjamin I, Ozawa T, Takamatsu K. Synchronization of circadian Per2 rhythms and HSF1-BMAL1:CLOCK interaction in mouse fibroblasts after short-term heat shock pulse. PLoS ONE. 2011;6(9):e24521. 10.1371/journal.pone.0024521. 21915348 10.1371/journal.pone.0024521 PMC3168500

[79] Lu J, Li H, Yu D, Zhao P, Liu Y. Heat stress inhibits the proliferation and differentiation of myoblasts and is associated with damage to mitochondria. Front Cell Dev Biol. 2023;11:1171506. 10.3389/fcell.2023.1171506. 37113771 10.3389/fcell.2023.1171506 PMC10126414

[80] Yang WR, Li BB, Hu Y, Zhang L, Wang XZ. Oxidative stress mediates heat-induced changes of tight junction proteins in porcine sertoli cells via inhibiting CaMKKβ-AMPK pathway. Theriogenology. 2020;142:104–13. 10.1016/j.theriogenology.2019.09.031. 31586867 10.1016/j.theriogenology.2019.09.031

[81] Tamaru T, Hattori M, Ninomiya Y, Kawamura G, Varès G, Honda K, et al. ROS stress resets circadian clocks to coordinate pro-survival signals. PLoS ONE. 2013;8(12):e82006. 10.1371/journal.pone.0082006. 24312621 10.1371/journal.pone.0082006 PMC3846904

[82] Zhang HY, Li KY, Wang YL, Wei CJ, Gao YX, Ren Z, et al. ROS regulates circadian rhythms by modulating Ezh2 interactions with clock proteins. Redox Biol. 2025;81:103526. 10.1016/j.redox.2025.103526. 39952198 10.1016/j.redox.2025.103526 PMC11875201

[83] Zhang X, Liu X, Wan F, You W, Tan X, Sheng Q, et al. Protective effect of resveratrol against hydrogen peroxide-induced oxidative stress in bovine skeletal muscle cells. Meat Sci. 2022;185:108724. 10.1016/j.meatsci.2021.108724. 34952489 10.1016/j.meatsci.2021.108724

[84] Song P, Zhao J, Li F, Zhao X, Feng J, Su Y, et al. Vitamin A regulates mitochondrial biogenesis and function through p38 MAPK-PGC-1α signaling pathway and alters the muscle fiber composition of sheep. J Anim Sci Biotechnol. 2024;15:18. 10.1186/s40104-023-00968-4. 38310300 10.1186/s40104-023-00968-4 PMC10838450

[85] Thanh LP, Wichasit N, Li Y, Batistel F, Tartrakoon W, Parys C, et al. Alterations in skeletal muscle abundance of protein turnover, stress, and antioxidant proteins during the periparturient period in dairy cows fed ethyl-cellulose rumen-protected methionine. J Dairy Sci. 2023;106(7):5127–45. 10.3168/jds.2022-23187. 37225585 10.3168/jds.2022-23187

[86] Reith RR, Sieck RL, Grijalva PC, Swanson RM, Fuller AM, Diaz DE, et al. Transcriptome analyses indicate that heat stress-induced inflammation in white adipose tissue and oxidative stress in skeletal muscle is partially moderated by zilpaterol supplementation in beef cattle. J Anim Sci. 2022;100(3):skac019. 10.1093/jas/skac019. 35079800 10.1093/jas/skac019 PMC8919836

[87] Chappuis S, Ripperger JA, Schnell A, Rando G, Jud C, Wahli W, et al. Role of the circadian clock gene Per2 in adaptation to cold temperature. Mol Metab. 2013;2(3):184–93. 10.1016/j.molmet.2013.05.002. 24049733 10.1016/j.molmet.2013.05.002 PMC3773826

[88] Niu L, Chen Q, Hua C, Geng Y, Cai L, Tao S, et al. Effects of chronic dexamethasone administration on hyperglycemia and insulin release in goats. J Anim Sci Biotechnol. 2018;9:26. 10.1186/s40104-018-0242-4. 10.1186/s40104-018-0242-4 PMC5855938 29568520

[89] Cheon S, Park N, Cho S, Kim K. Glucocorticoid-mediated Period2 induction delays the phase of circadian rhythm. Nucleic Acids Res. 2013;41(12):6161–74. 10.1093/nar/gkt307. 23620290 10.1093/nar/gkt307 PMC3695510

[90] Xu Z, Liu J, You W, Wang Y, Shan T. Cold exposure induces nuclear translocation of CRTC3 in brown adipose tissue. J Cell Biochem. 2019;120(6):9138–46. 10.1002/jcb.28189. 30506739 10.1002/jcb.28189

[91] Chen L, Qin Y, Liu B, Gao M, Li A, Li X, et al. PGC-1α-mediated mitochondrial quality control: molecular mechanisms and implications for heart failure. Front Cell Dev Biol. 2022;10:871357. 10.3389/fcell.2022.871357. 35721484 10.3389/fcell.2022.871357 PMC9199988

[92] Raza GS, Sodum N, Kaya Y, Herzig KH. Role of circadian transcription factor Rev-Erb in metabolism and tissue fibrosis. Int J Mol Sci. 2022;23(21):12954. 10.3390/ijms232112954. 36361737 10.3390/ijms232112954 PMC9655416

[93] Buhr ED, Yoo SH, Takahashi JS. Temperature as a universal resetting cue for mammalian circadian oscillators. Science. 2010;330(6002):379–85. 10.1126/science.1195262. 20947768 10.1126/science.1195262 PMC3625727

[94] Fuchs B, Sørheim KM, Chincarini M, Brunberg E, Stubsjøen SM, Bratbergsengen K, et al. Heart rate sensor validation and seasonal and diurnal variation of body temperature and heart rate in domestic sheep. Vet Anim Sci. 2019;8:100075. 10.1016/j.vas.2019.100075. 32734092 10.1016/j.vas.2019.100075 PMC7386703

[95] Guo Y, Wang QJ, Zhang KH, Yao CY, Huang J, Li Q, et al. Night-restricted feeding improves locomotor activity rhythm and modulates nutrient utilization to accelerate growth in rabbits. FASEB J. 2021;35(1):e21166. 10.1096/fj.202001265RR. 33184921 10.1096/fj.202001265RR

[96] Dai JM, Yu MX, Shen ZY, Guo CY, Zhuang SQ, Qiu XS. Leucine promotes proliferation and differentiation of primary preterm rat satellite cells in part through mTORC1 signaling pathway. Nutrients. 2015;7(5):3387–400. 10.3390/nu7053387. 26007333 10.3390/nu7053387 PMC4446757

[97] Drummond MJ, Glynn EL, Fry CS, Dhanani S, Volpi E, Rasmussen BB. Essential amino acids increase microRNA-499, -208b, and -23a and downregulate myostatin and myocyte enhancer factor 2C mRNA expression in human skeletal muscle. J Nutr. 2009;139(12):2279–84. 10.3945/jn.109.112797. 19828686 10.3945/jn.109.112797 PMC2777476

[98] Zhang Y, Yu B, He J, Chen D. From nutrient to microRNA: a novel insight into cell signaling involved in skeletal muscle development and disease. Int J Biol Sci. 2016;12(10):1247–61. 10.7150/ijbs.16463. 27766039 10.7150/ijbs.16463 PMC5069446

[99] Leprivier G, Rotblat B. How does mTOR sense glucose starvation? AMPK is the usual suspect. Cell Death Discov. 2020;6:27. 10.1038/s41420-020-0260-9. 32351714 10.1038/s41420-020-0260-9 PMC7176732

[100] Li Z, Fu B, Wei A, Wu Y, Huang M, Zhang E, et al. D-Glucosamine induces circadian phase delay by promoting BMAL1 degradation through AMPK/mTOR pathway. Life Sci. 2023;325:121765. 10.1016/j.lfs.2023.121765. 37169147 10.1016/j.lfs.2023.121765

[101] Chen J, Xiang J, Zhou M, Huang R, Zhang J, Cui Y, et al. Dietary timing enhances exercise by modulating fat-muscle crosstalk via adipocyte AMPKα2 signaling. Cell Metab. 2025;37(6):1364-80.e6. 10.1016/j.cmet.2025.02.007. 40088888 10.1016/j.cmet.2025.02.007

[102] Zhang Z, Liu BB, Ding SZ. External cues as transducers of peripheral tissue-specific molecular clocks to regulate systemic circadian rhythms and metabolism. FASEB J. 2025;39(17):e71011. 10.1096/fj.202501289R. 40917013 10.1096/fj.202501289R

[103] Rodrigues LGF, de Araujo LD, Roa SLR, Bueno AC, Uchoa ET, Antunes-Rodrigues J, et al. Restricted feeding modulates peripheral clocks and nutrient sensing pathways in rats. Arch Endocrinol Metab. 2021;65(5):549–61. 10.20945/2359-3997000000407. 34591411 10.20945/2359-3997000000407 PMC10528573

[104] Duszka K, Wahli W. Peroxisome proliferator-activated receptors as molecular links between caloric restriction and circadian rhythm. Nutrients. 2020;12(11):3476. 10.3390/nu12113476. 33198317 10.3390/nu12113476 PMC7696073

[105] Aoyama S, Kim HK, Hirooka R, Tanaka M, Shimoda T, Chijiki H, et al. Distribution of dietary protein intake in daily meals influences skeletal muscle hypertrophy via the muscle clock. Cell Rep. 2021;36(1):109336. 10.1016/j.celrep.2021.109336. 34233179 10.1016/j.celrep.2021.109336

[106] Yu K, Matzapetakis M, Horvatić A, Terré M, Bach A, Kuleš J, et al. Metabolome and proteome changes in skeletal muscle and blood of pre-weaning calves fed leucine and threonine supplemented diets. J Proteomics. 2020;216:103677. 10.1016/j.jprot.2020.103677. 32028040 10.1016/j.jprot.2020.103677

[107] Lv X, Jiang A, Hua J, Liu Z, Yan Q, Tang S, et al. Long-term leucine supplementation increases body weight in goats by controlling appetite and muscle protein synthesis under protein-restricted conditions. Anim Nutr. 2025;20:404–18. 10.1016/j.aninu.2024.09.005. 40034461 10.1016/j.aninu.2024.09.005 PMC11872668

[108] Wu X, Xie C, Guo X, Long C, Zhang T, Gao T, et al. A maternal two-meal feeding sequence with varying crude protein affects milk lipid profile in a sow-piglet model. Sci Rep. 2017;7:13742. 10.1038/s41598-017-14188-0. 10.1038/s41598-017-14188-0 PMC5653795 29062061

[109] Wang QJ, Guo Y, Yao CY, Zhang KH, Li Q, Shan CH, et al. Loss of diurnal behavioral rhythms and impaired lipid metabolism in growing pigs with mistimed feeding. FASEB J. 2021;35(11):e21972. 10.1096/fj.202100768R. 34613642 10.1096/fj.202100768R

[110] Wang H, Zhang H, Su Y. New insights into the diurnal rhythmicity of gut microbiota and its crosstalk with host circadian rhythm. Animals. 2022;12(13):1677. 10.3390/ani12131677. 35804575 10.3390/ani12131677 PMC9264800

[111] Hao Y, Wang W, Li M, Choi Y, Zhou M, Wang Y, et al. Microbial diurnal rhythmicity in the rumen fluid impacted by feeding regimes and exogenous microbiome providing novel mechanisms regulating dynamics of the rumen microbiome. Microbiome. 2025;13:142. 10.1186/s40168-025-02134-6. 10.1186/s40168-025-02134-6 PMC12168421 40524215

[112] Pekmez CT, Dragsted LO, Brahe LK. Gut microbiota alterations and dietary modulation in childhood malnutrition - the role of short chain fatty acids. Clin Nutr. 2019;38(2):615–30. 10.1016/j.clnu.2018.02.014. 29496274 10.1016/j.clnu.2018.02.014

[113] Zhu J, Peng F, Yang H, Luo J, Zhang L, Chen X, et al. Probiotics and muscle health: the impact of *Lactobacillus* on sarcopenia through the gut-muscle axis. Front Microbiol. 2025;16:1559119. 10.3389/fmicb.2025.1559119. 40160272 10.3389/fmicb.2025.1559119 PMC11952772

[114] Chen L, Shi Y, Li J, Shao C, Ma S, Shen C, et al. Dietary bile acids improve breast muscle growth in chickens through FXR/IGF2 pathway. Poult Sci. 2024;103(2):103346. 10.1016/j.psj.2023.103346. 38128457 10.1016/j.psj.2023.103346 PMC10776637

[115] Mancin L, Wu GD, Paoli A. Gut microbiota–bile acid–skeletal muscle axis. Trends Microbiol. 2023;31(3):254–69. 10.1016/j.tim.2022.10.003. 36319506 10.1016/j.tim.2022.10.003

[116] Li L, Li J, Liu Z, Jin Z, Wang M, Wu Y, et al. Effects of supplementing bile acids on the production performance, fatty acid and bile acid composition, and gut microbiota in transition dairy cows. J Anim Sci Biotechnol. 2025;16:83. 10.1186/s40104-025-01207-8. 40506720 10.1186/s40104-025-01207-8 PMC12160099

[117] Ouyang J, Wang M, Bu D, Ma L, Liu F, Xue C, et al. Ruminal microbes exhibit a robust circadian rhythm and are sensitive to melatonin. Front Nutr. 2021;8:760578. 10.3389/fnut.2021.760578. 34760910 10.3389/fnut.2021.760578 PMC8573100

[118] Liu B, Fan L, Wang Y, Wang H, Yan Y, Chen S, et al. Gut microbiota regulates host melatonin production through epithelial cell MyD88. Gut Microbes. 2024;16(1):2313769. 10.1080/19490976.2024.2313769. 38353638 10.1080/19490976.2024.2313769 PMC10868534

[119] Ducharme JB, Fennel ZJ, McKenna ZJ, Nava RC, Deyhle MR. Stimulated myotube contractions regulate membrane-bound and soluble TLR4 to prevent LPS-induced signaling and myotube atrophy in skeletal muscle cells. Am J Physiol Cell Physiol. 2023;325(1):C300–13. 10.1152/ajpcell.00007.2023. 37335026 10.1152/ajpcell.00007.2023

[120] Mukherji A, Kobiita A, Ye T, Chambon P. Homeostasis in intestinal epithelium is orchestrated by the circadian clock and microbiota cues transduced by TLRs. Cell. 2013;153(4):812–27. 10.1016/j.cell.2013.04.020. 23663780 10.1016/j.cell.2013.04.020

[121] Ono Y, Sakamoto K. Lipopolysaccharide inhibits myogenic differentiation of C2C12 myoblasts through the Toll-like receptor 4-nuclear factor-κB signaling pathway and myoblast-derived tumor necrosis factor-α. PLoS ONE. 2017;12(7):e0182040. 10.1371/journal.pone.0182040. 28742154 10.1371/journal.pone.0182040 PMC5524356

[122] Moretti CH, Grasset E, Zhu J, Yang G, Olofsson LE, Khan MT, et al. Identification of human gut bacteria that produce bioactive serotonin and promote colonic innervation. Cell Rep. 2025;44(10):116434. 10.1016/j.celrep.2025.116434. 41118765 10.1016/j.celrep.2025.116434

[123] Aoki N, Watanabe H, Okada K, Aoki K, Imanishi T, Yoshida D, et al. Involvement of 5-HT3 and 5-HT4 receptors in the regulation of circadian clock gene expression in mouse small intestine. J Pharmacol Sci. 2014;124(2):267–75. 10.1254/jphs.13253FP. 24492464 10.1254/jphs.13253fp

[124] Fefeu M, Blatzer M, Kneppers A, Briand D, Rocheteau P, Haroche A, et al. Serotonin reuptake inhibitors improve muscle stem cell function and muscle regeneration in male mice. Nat Commun. 2024;15:6457. 10.1038/s41467-024-50220-4. 10.1038/s41467-024-50220-4 PMC11291725 39085209

[125] Montagner A, Korecka A, Polizzi A, Lippi Y, Blum Y, Canlet C, et al. Hepatic circadian clock oscillators and nuclear receptors integrate microbiome-derived signals. Sci Rep. 2016;6:20127. 10.1038/srep20127. 26879573 10.1038/srep20127 PMC4754633

[126] Lahiri S, Kim H, Garcia-Perez I, Reza MM, Martin KA, Kundu P, et al. The gut microbiota influences skeletal muscle mass and function in mice. Sci Transl Med. 2019;11(502):eaan5662. 10.1126/scitranslmed.aan5662. 31341063 10.1126/scitranslmed.aan5662 PMC7501733

[127] Dou L, Liu C, Chen X, Yang Z, Hu G, Zhang M, et al. Supplemental *Clostridium butyricum* modulates skeletal muscle development and meat quality by shaping the gut microbiota of lambs. Meat Sci. 2023;204:109235. 10.1016/j.meatsci.2023.109235. 37301103 10.1016/j.meatsci.2023.109235

[128] Mayeuf-Louchart A, Staels B, Duez H. Skeletal muscle functions around the clock. Diabetes Obes Metab. 2015;17(1):39–46. 10.1111/dom.12517. 26332967 10.1111/dom.12517

[129] Kirchner H, Weisner L, Wilms B. When should I run—the role of exercise timing in metabolic health. Acta Physiol (Oxf). 2023;237(4):e13953. 10.1111/apha.13953. 10.1111/apha.13953 36815281

[130] Mansingh S, Handschin C. Time to train: the involvement of the molecular clock in exercise adaptation of skeletal muscle. Front Physiol. 2022;13:902031. 10.3389/fphys.2022.902031. 35547572 10.3389/fphys.2022.902031 PMC9081842

[131] Viggars MR, Berko HE, Hesketh SJ, Wolff CA, Gutierrez-Monreal MA, Martin RA, et al. Skeletal muscle BMAL1 is necessary for transcriptional adaptation of local and peripheral tissues in response to endurance exercise training. Mol Metab. 2024;86:101980. 10.1016/j.molmet.2024.101980. 38950777 10.1016/j.molmet.2024.101980 PMC11294728

[132] Healy KL, Morris AR, Liu AC. Circadian synchrony: sleep, nutrition, and physical activity. Front Netw Physiol. 2021;1:732243. 10.3389/fnetp.2021.732243. 35156088 10.3389/fnetp.2021.732243 PMC8830366

[133] Peek CB, Levine DC, Cedernaes J, Taguchi A, Kobayashi Y, Tsai SJ, et al. Circadian clock interaction with HIF1α mediates oxygenic metabolism and anaerobic glycolysis in skeletal muscle. Cell Metab. 2017;25(1):86–92. 10.1016/j.cmet.2016.09.010. 27773696 10.1016/j.cmet.2016.09.010 PMC5226863

[134] Zhao M, Zhang X, Chen Y, Ren C, Sun Y, Wang P, et al. Stall-feeding of sheep on restricted grazing: effects on performance and serum metabolites, ruminal fermentation, and fecal microbiota. Animals. 2023;13(16):2644. 10.3390/ani13162644. 37627436 10.3390/ani13162644 PMC10451354

[135] Li Z, Zhao X, Jian L, Wang B, Luo H. Rumen microbial-driven metabolite from grazing lambs potentially regulates body fatty acid metabolism by lipid-related genes in liver. J Anim Sci Biotechnol. 2023;14:39. 10.1186/s40104-022-00823-y. 36879349 10.1186/s40104-022-00823-y PMC9990365

[136] Marquez DC, Paulino MF, Rennó LN, Villadiego FC, Ortega RM, Moreno DS, et al. Supplementation of grazing beef cows during gestation as a strategy to improve skeletal muscle development of the offspring. Animal. 2017;11(12):2184–92. 10.1017/S1751731117000982. 28571587 10.1017/S1751731117000982

